# Suppression of tumor angiogenesis by metformin treatment *via* a mechanism linked to targeting of HER2/HIF-1α/VEGF secretion axis

**DOI:** 10.18632/oncotarget.6373

**Published:** 2015-11-02

**Authors:** Jichang Wang, Guangyue Li, Yaochun Wang, Shouching Tang, Xin Sun, Xuefei Feng, Yan Li, Gang Bao, Pingping Li, Xiaona Mao, Maode Wang, Peijun Liu

**Affiliations:** ^1^ Center for Translational Medicine, the First Affiliated Hospital of Xi'an Jiaotong University, Xi'an, Shaanxi Province, 710061, P.R.China; ^2^ Department of Neurosurgery, the First Affiliated Hospital of Xi'an Jiaotong University, Xi'an, Shaanxi Province, 710061, P.R.China; ^3^ Department of Vascular Surgery, the First Affiliated Hospital of Xi'an Jiaotong University, Xi'an, Shaanxi Province, 710061, P.R.China; ^4^ Department of Thoracic Surgery and Oncology, the First Affiliated Hospital of Xi'an Jiaotong University, Xi'an, Shaanxi Province, 710061, China; ^5^ Breast Cancer Program and Interdisciplinary Translational Research Team, Georgia Regents University Cancer Center, Augusta, Georgia, 30912, United States of America; ^6^ Tianjin Medical University Cancer Institute and Hospital, Tianjin, 300060, China; ^7^ Department of Science and Technology, the First Affiliated Hospital of Xi'an Jiaotong University, Xi'an, Shaanxi Province, 710061, P.R.China

**Keywords:** metformin, anti-angiogenesis, HER2, heregulin-β1, HIF-1α-VEGF signaling

## Abstract

Anti-angiogenesis is currently considered as one of the major antitumor strategies for its protective effects against tumor emergency and later progression. The anti-diabetic drug metformin has been demonstrated to significantly inhibit tumor angiogenesis based on recent studies. However, the mechanism underlying this anti-angiogenic effect still remains an enigma. In this study, we investigated metformin-induced inhibitory effect on tumor angiogenesis *in vitro* and *in vivo*. Metformin pretreatment significantly suppressed tumor paracrine signaling-induced angiogenic promotion even in the presence of heregulin (HRG)-β1 (a co-activator of HER2) pretreatment of HER2^+^ tumor cells. Similar to that of AG825, a specific inhibitor of HER2 phosphorylation, metformin treatment decreased both total and phosphorylation (Tyr 1221/1222) levels of HER2 protein and significantly reduced microvessel density and the amount of Fitc-conjugated Dextran leaking outside the vessel. Furthermore, our results of VEGF-neutralizing and -rescuing tests showed that metformin markedly abrogated HER2 signaling-induced tumor angiogenesis by inhibiting VEGF secretion. Inhibition of HIF-1α signaling by using RNAi or YC-1, a specific inhibitor of HIF-1α synthesis, both completely diminished mRNA level of VEGF and greatly inhibited endothelial cell proliferation promoted by HER2^+^ tumor cell-conditioned medium in both the absence and presence of HRG-β1 pretreatment. Importantly, metformin treatment decreased the number of HIF-1α nucleus positive cells in 4T1 tumors, accompanied by decreased microvessel density. Our data thus provides novel insight into the mechanism underlying the metformin-induced inhibition of tumor angiogenesis and indicates possibilities of HIF-1α-VEGF signaling axis in mediating HER2-induced tumor angiogenesis.

## INTRODUCTION

Angiogenesis, the formation of nascent blood vessels from preexisting vasculature, is a fundamental event in the process of tumor growth. However, this physiological process can be tamed by tumor cells mediating distant metastasis or dissemination [1, 2]. From the early stage of tumorigenesis, tumor cells produce a large number of pro-angiogenic factors to form a nascent vascular network that subsequently penetrates deep into the tumor [3]. This kind of vasculature induced by tumor cells is essential for tumor growth, because it is capable of transporting oxygen and nutrients into tumor, thus supporting tumor growth and progression. However, tumor cells migrating into the internal lumen of vessels can also be transported to nearby tissues or distant organs, thus forming new tumor lesions. It has been recently reported that, therapies aimed at targeting tumor angiogenesis not only suppressed tumor growth, but also reduced the risk of tumor metastasis and led to better survival rates [4]. Therefore, anti-angiogenesis, an antitumor strategy introduced by Judah Folkman nearly four decades ago, is currently considered as a major antitumor modality [4, 5].

Metformin, a biguanide derivate, is the most widely prescribed drug for medical management of types II diabetes (T2D) and in the recent guidelines of the American Diabetes Association (ADA), it is recommended as a first-line oral treatment for the disease [6]. Since the last decade, metformin has attracted attentions again for its significant antitumor activities [7–9]. The recent evidence has accumulated suggesting that metformin has the potential to impede *in vitro* angiogenesis mediated by human umbilical vein endothelial cells (HUVECs) [10, 11]. As was observed in other *in vivo* models, metformin greatly hindered angiogenesis in matrigel pellets and significantly decreased the microvessel density (MVD) in solid tumors [9, 12]. This evidence has revealed the potential of metformin in suppressing tumor angiogenesis. Although recent studies suggest that metformin may actively target components of the microenvironment [9], in fact, paracrine mechanisms mediated by angiogenic factors released by tumor cells play an essential role in promoting angiogenesis in the process of tumor development [3]. However, the underlying mechanisms of whether or how metformin inhibits tumor angiogenesis by affecting tumor secretion of angiogenic cytokines secretion remains unknown.

Human epidermal growth factor receptor-2 (HER2), a member of epidermal growth factor receptor (EGFR) family, is overexpressed in about 25% of invasive breast cancers and its expression is positively correlated with vascular endothelial growth factor (VEGF)-associated high vascularity of within solid tumor [13, 14]. Although hypoxia inducible factor 1α (HIF-1α) has been shown to directly regulate VEGF expression and secretion, it is still largely unknown whether, or to what extent, HIF-1α is involved in HER2-induced VEGF up-regulation [15]. Indeed, HIF-1α has been demonstrated to be important for HER2 signaling-induced tumor progression and angiogenesis [16]. In this study, we explored if the HIF-1α-VEGF secretion axis was involved in metformin-induced angiogenic abrogation of cancer cells with highly phosphorylated HER2. To further study the effects of metformin on suppressing HER2 signaling-associated angiogenesis, recombinant heregulin (HRG)-β1, a co-activator of HER2, and AG825, a specific inhibitor of HER2 phosphorylation [17], were used for treatment of HER2^+^ cell lines. Through decreasing the production of HER2 protein, metformin induced a similar effects as AG825 on suppressing HER2 phosphorylation, thus restraining the activity of HIF-1α-VEGF signaling axis and suppressing tumor angiogenesis *in vivo*.

## RESULTS

### Protein expressions of HER2, HIF-1α, and VEGF in various breast cell lines

**Figure 1:**
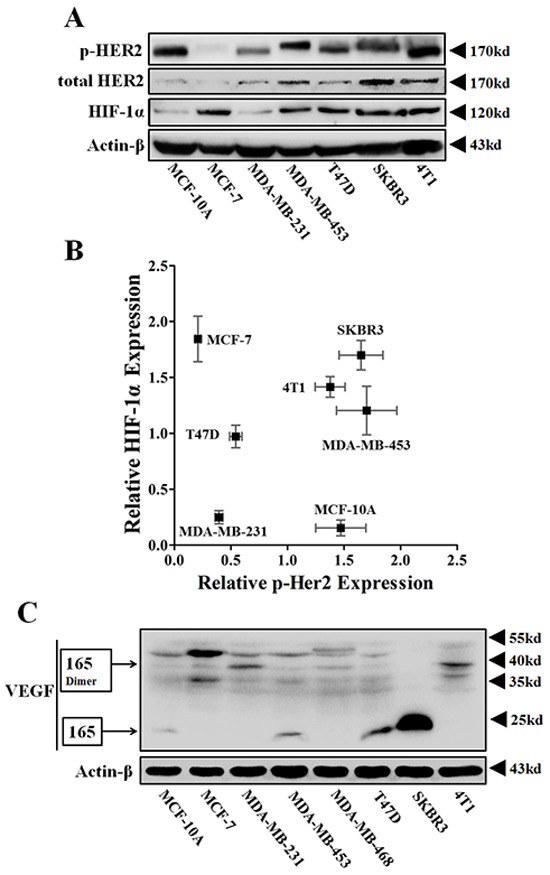
The protein expressions of p-HER2 (Tyr 1221/1222), total HER2, HIF-1α and VEGFA of various breast cancer cells **A.** Immunoblotting for protein expressions of total HER2, p-HER2 and HIF-1α in normoxia. 100 μg protein per lane. **B.** Representative scatter-plot revealing the relationship between the protein levels of p-HER2 and HIF-1α in various breast cancer cells (*n* = 3). **C.** Immunoblotting for VEGFA expression in various breast cell lines. The main VEGFA isoform, VEGF165, and its homodimers were respectively detected at 23 kD and 38 kD.

We first detected the protein expressions of phosphos-HER2, HIF-1α and VEGFA in eight breast cell lines. Because EGF was added to the culture medium, HER2 was greatly phosphorylated in MCF-10A (Figure 1A), a normal mammary epithelial cell. However, the HIF-1α-VEGF signaling axis was not apparently activated in these cells (Figure 1A–1C). In the tri-negative cell line, MDA-MB-231, low levels of phospho-HER2 were accompanied by low protein levels of HIF-1α and VEGF (Figure 1A–1C). Inversely, HIF-1α-VEGF signaling was highly activated in cancer cell lines (MDA-MB-453, SKBR3 and 4T1) with highly phosphorylated HER2 protein (Figure 1A–1C). Besides, HIF-1α was also highly expressed in T47D and MCF-7 cells, which have been generally recognized as the estrogen receptor (ER) (+)/ progestin receptor (PR) (+) cell lines (Figure 1A and 1B). These data indicated that HER2 might be an oncogene that was closely correlated with the expressions of HIF-1α and VEGF, like ER and PR.

### Metformin inhibited HER2^+^ tumor angiogenesis via a paracrine mechanism

**Figure 2:**
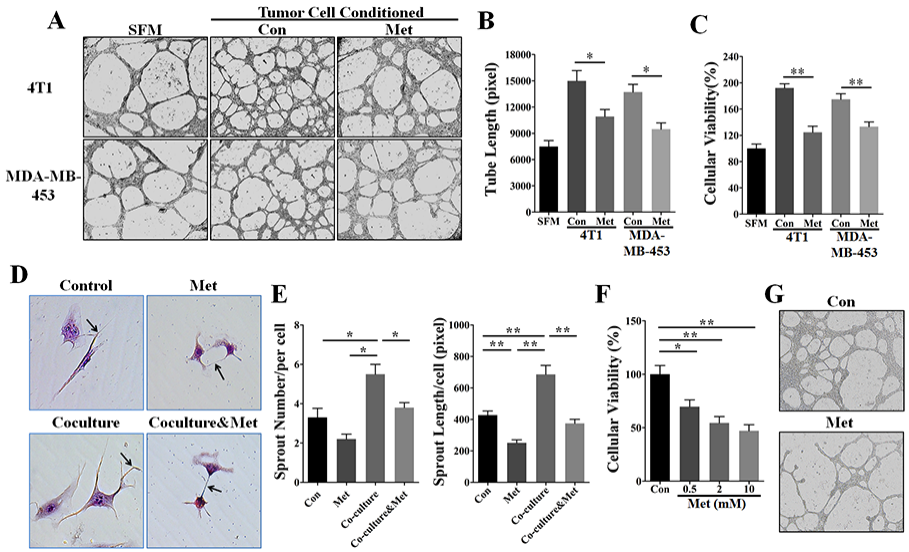
Metformin inhibited HER2+ tumor angiogenesis by a paracrine related mechanism HUVECs were cultured in serum-free medium (SFM) or 75% tumor cell-conditioned medium (TCM) of 4T1 or MDA-MB-453 cells. **A.** Metformin (10 mM) pretreatment inhibited HUVEC-mediated tube formation promoted by the TCM of 4T1 and MDA-MB-453 cells. 200X. **B.** Quantification of the tube length produced by HUVECs (*n* = 5). **C.** Metformin pretreatment (10 mM) abrogated the promotion of HUVEC proliferation induced by the TCM of 4T1 and MDA-MB-453 cells (*n* = 5). **D.** HUVEC were co-cultured with MDA-MB-453 cells using a transwell cell culture system (pore size: 0.4 μm) that did not allow for direct cell-cell contact. Black arrows indicate endothelial cells sprouts. 400X. **E.** Quantification of the number and length (pixel) of HUVEC-mediated vascular sprouts (*n* = 5, respectively). **F.** Metformin induced direct inhibition of HUVEC proliferation in a dose-dependent manner. *p* = 0.0023, one-way ANOVA. **G.** HUVECs inoculated onto the matrigel were directly treated with metformin (10 mM) for 24 h. 100X. All quantitative data are presented as mean ± S.E.M. *p < 0.05, **p < 0.01.

We then investigated the effect of metformin on suppressing the angiogenesis promoted by the paracrine signaling of HER2^+^ tumor cells. Tumor cell conditioned medium (TCM) from 4T1 and MDA-MB-453 cells pre-treated with or without metformin was used to culture endothelial cells (ECs). As shown in tube formation assay results (Figure 2A and 2B), ECs (HUVEC and HMEC-1) treated with 4T1 or MDA-MB-453 TCM developed longer tube structures than those cultured in serum-free medium (SFM). However, tumor cells were pre-treated with 10 mM metformin, the tube length produced by HUVECs was significantly reduced to a level slightly higher than that in cells treated with SFM (Figure 2A and Supplementary Figure S1). Since EC proliferation is an essential element in the process of angiogenesis, we next investigated if metformin affected TCM-promoted EC proliferation. As shown, HUVECs proliferation-promoted by TCM of both MDA-MB-453 and 4T1 cells was significantly abrogated by metformin pretreatment via a time-dependent manner (Figure 2C and Supplementary Figure S2). These data indicated that metformin potentially repress paracrine signaling-mediated angiogenesis of HER2^+^ tumor cells.

### Metformin suppressed vascular sprouting ability of EC in a co-culture system

Since tumor angiogenesis results from the interaction of cancer cells with endothelial cells for the most part, we next utilized an indirect coculture system to simulate the *in vivo* tumor angiogenesis, aiming to investigate metformin-induced anti-angiogenic effect via affecting cancer-endothelial cells interaction. In this system, cancer cells can't directly contact with ECs, and the molecular and drug particles can freely diffuse. In the absence of co-culture, metformin directly weakened the vascular sprouting ability of HUVECs (Figure 2D and 2E), suggesting metformin has a direct effect on suppressing EC function. In addition, HUVECs generated more and longer vascular sprouts in co-culture with MDA-MB-453 cells than those that were not co-cultured. Importantly, co-culture-associated increases of number and length of vascular sprouts were significantly abrogated by metformin treatment. To further verify the direct effect of metformin, we focused on the changes of HUVECs viability and tube formation ability. As shown in (Figure 2F and 2G), metformin significantly suppressed HUVECs proliferation and tube formation ability. Taken together, our data demonstrated the dual effects of metformin on suppressing tumor angiogenesis: directly restraining the ECs function and indirectly impeding tumor paracrine signaling.

### HER2 signaling was involved in metformin-induced angiogenic suppression in 4T1 breast cancer model

**Figure 3:**
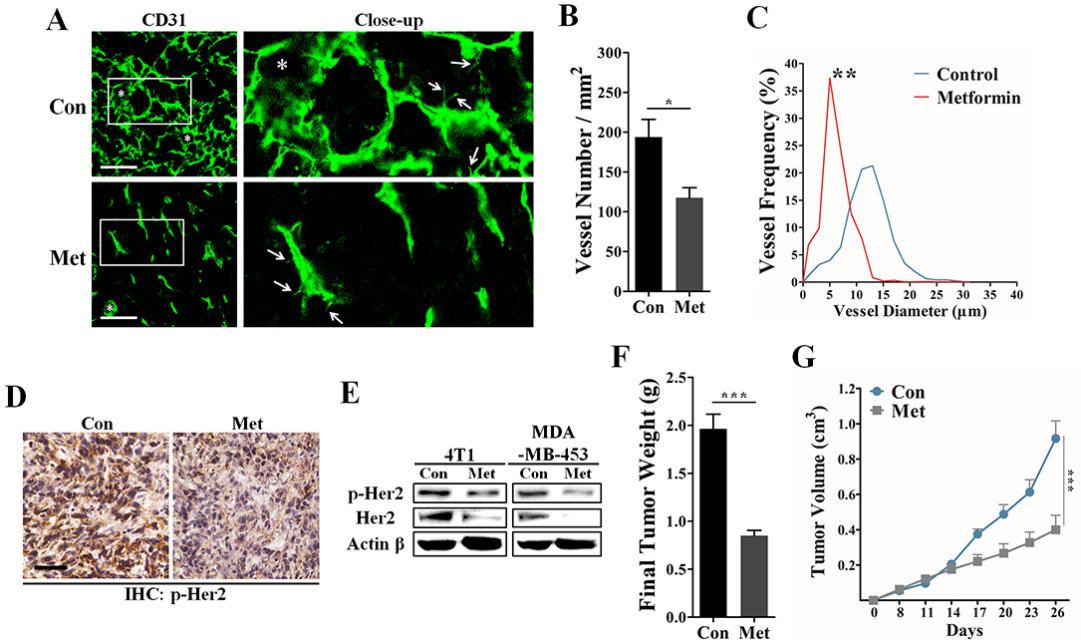
Inhibitory effects of metformin on tumor angiogenesis and HER2 activity In the 4T1 breast carcinoma model, the BALB/C mice were orally administrated with metformin (200 mg/kg • day) or drinking water (control) for 14 days after the average tumor volume reached 100 mm^3^. **A.** Representative 3D-reconstruction image for detecting CD31^+^ vessels in 4T1 tumors. White stars and arrows indicate the dilated vessels and vascular sprouts, respectively. Scale, 50 μm. **B.** Quantification of microvessel density in 4T1 tumor sections (*n* = 8). **C.** Representative image showing the frequency distribution of vessel diameter in 4T1 tumors (*n* = 8). **D.** Immunohistochemical staining for protein expression of phospho-HER2 (Tyr 1221/1222) in 4T1 tumors. Scale, 50 μm. **E.** Immunoblotting for both total and phosphorylated levels of HER2 protein in 4T1 and MDA-MB-453 cell cells untreated or treated with 10 mM metformin. 80 μg protein per lane. Quantification of the final weight **F.** and growth curve **G.** of 4T1 tumors from mice untreated or treated with metformin (200 mg/kg • day). All data is presented as mean ± S.E.M. *p < 0.05, **p < 0.01, ***p < 0.001.

To investigate the effect of metformin on suppressing *in vivo* tumor angiogenesis, we next used the transplanted murine 4T1 cancer model, which is poorly immunogenic and highly vascularized. Consistent with its high VEGF expression, 4T1 tumor was characterized by high MVD, vascular leakage and intense blood vessel leakage (Figure 3A). Immunofluorescent results demonstrated that metformin treatment (200 mg/kg • day) greatly decreased the MVD and reduced the length of vascular sprout in 4T1 tumors (Figure 3A and 3B). Because vascular dilation has been demonstrated to be one important hallmark of tumor vasculature, we next focused on the effect of metformin on decreasing the diameter of tumor vessels. In metformin-treated samples, large-diameter vessels typically seen in 4T1 tumors were rarely detectable and the tumor vessel size was smaller (Figure 3A and 3C).

To study whether HER2 signaling was involved in metformin-induced angiogenesis suppression, we next detected the change of HER2 expression using IHC and western blotting experiments. As shown in Figure 3D and Supplementary Figure S3, 4T1 tumor tissue from metformin treated mice showed a lower level of phospho-HER2 (Tyr 1221/1222). In addition, metformin greatly decreased the phosphorylation level of HER2 protein *in vitro* via a time-dependent manner (Supplementary Figure S4). Consistent with our histological results, metformin apparently decreased both total and phosphorylated levels of HER2 proteins in both 4T1 and MDA-MB-453 cells (Figure 3E), while the mRNA level was not obviously affected (Supplementary Figure S5). Since HER2 signaling has a strong effect on promoting tumor proliferation, we next focused on the effect of metformin on inhibiting tumor growth *in vivo*. We found that metformin treatment induced a significant inhibition of 4T1 tumor growth *in vivo* (Figure 3F and 3G). Altogether, our data suggest HER2 may be an important molecular target of metformin in mediating inhibition of tumor growth and angiogenesis.

### Metformin abrogated HER2-induced angiogenic promotion involving up-regulated VEGF expression

**Figure 4:**
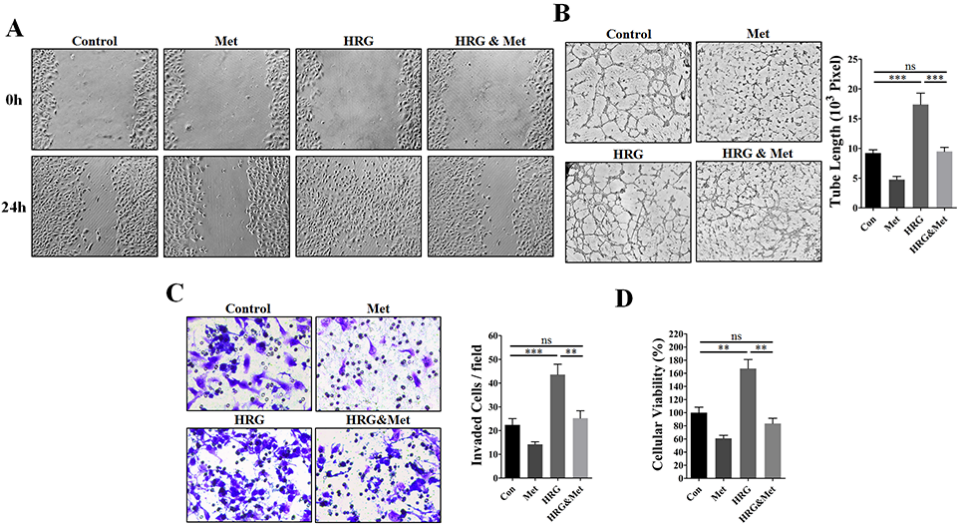
Metformin abrogated HRG-β1-HER2 signaling-induced angiogenic promotion *in vitro* HUVECs were cultured in the presence or absence of 75% TCM of MDA-MB-453 cells pre-treated with human recombinant HRG-β1 (50 ng/ml) or metformin (10 mM). **A.** Representative images showing wound-healing migration of HUVECs treated with the TCM of MDA-MB-453 cells. 100X. **B.** Representative images showing matrigel tube formation of HUVECs treated with TCM of MDA-MB-453 and the quantification of the tube length (*n* = 5, respectively). 200X. **C.** Representative images showing the invasion ability of HUVECs treated with TCM of MDA-MB-453 and quantification of the number of invaded cells per field (*n* = 5, respectively). 400X. **D.** Metformin pretreatment (10 mM) inhibited HUVEC proliferation promoted by tumor cell-conditioned medium from 4T1 and MDA-MB-453 cells pre-treated with HRG-β1 (50 ng/ml) (*n* = 5). All data is presented as mean ± S.E.M. *P < 0.05, **P < 0.01.

Since endogenous HRGs has been generally recognized for their crucial roles in promoting angiogenesis by enhancing HER2 signaling [18–21], we thus used human recombinant HRG-β1 as a HER2 activator for further investigation. As shown in Figure 4A, HUVECs cultured in TCM from HRG-β1 pretreated MDA-MB-453 cells showed a stronger migration capacity than those cultured in TCM from MDA-MB-453 cells with no pretreatment. Moreover, metformin pretreatment greatly abrogated HRG-β1-induced promotion of invasion, proliferation and tube formation abilities of HUVECs (Figure 4B–4D). Even in the absence of HRG-β1 pretreatment, MDA-MB-453 TCM induced-enhancement of EC-mediated angiogenesis was also greatly impaired.

**Figure 5:**
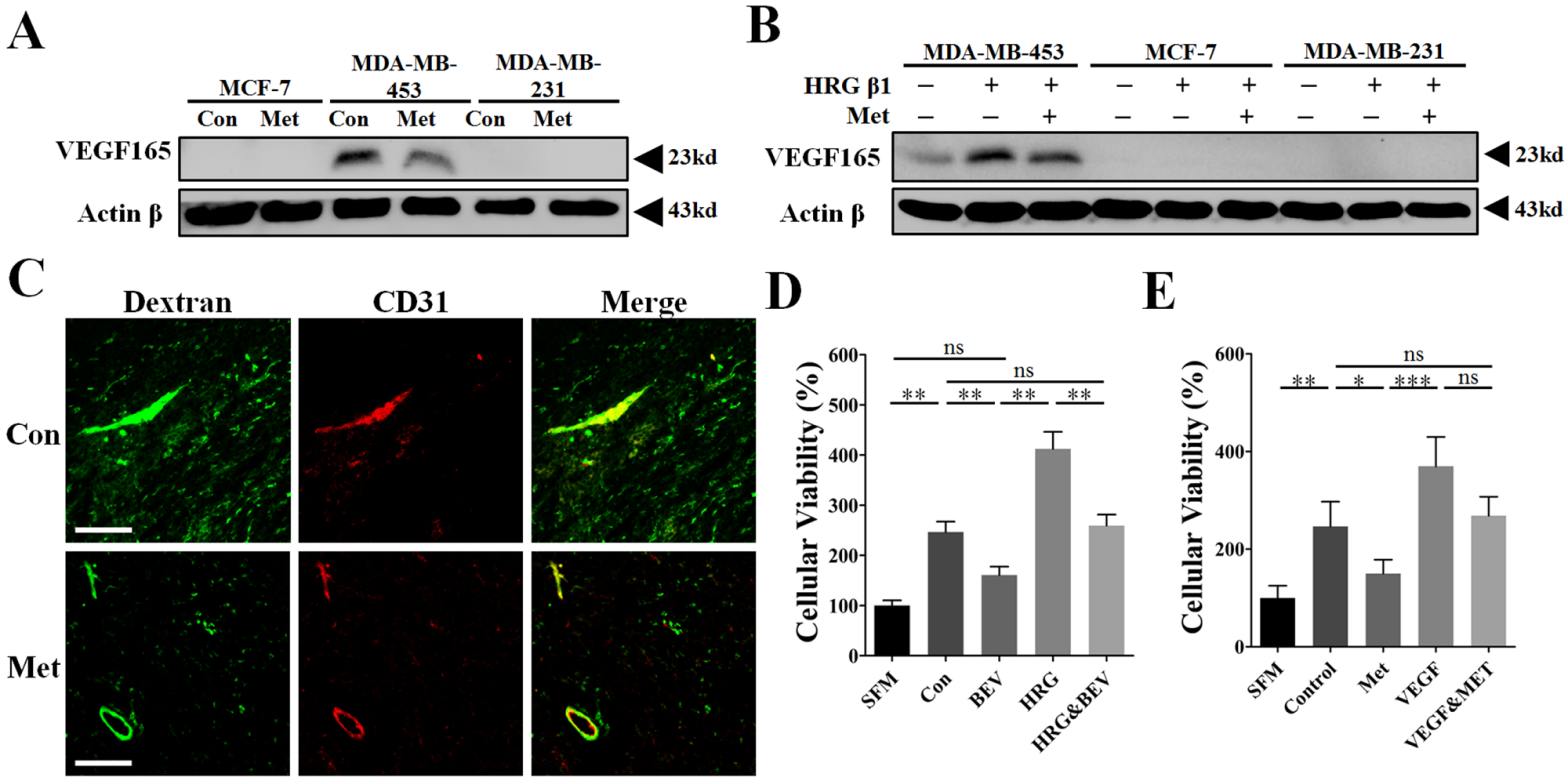
Inhibition of VEGFA signaling was involved in the mechanism of metformin-induced anti-angiogenesis and reduction of vessel leakage **A, B.** Immunoblotting for protein expression of VEGF_165_ in MCF-7, MDA-MB-231 and MDA-MB-453 cells untreated or treated with metformin, HRG-β1 or the combined treatment for 24 h. 50 μg protein per lane. **C.** Thirty minutes before mice were sacrificed, 100 mg/kg Fitc-conjugated Dextran (70 kD) in 100 μl was intravenously injected. CD31. Fitc signaling (Green) outside the boundary of TRITC signaling (Red) was considered as the dextran leaking outside the tumor vessel. **D.** The tumor cell-conditioned medium (TCM) of MDA-MB-453 cells with or without HRG-β1 pretreatment was pre-incubated with bevacizumab (250 μg/ml) for 1 h. Human umbilical endothelial cells (HUVECs) were then cultured with the mixture of TCM and BEV and finally the cellular viability was determined (*n* = 6). **E.** Human recombinant VEGFA (10 ng/ml) was first added to the TCM of MDA-MB-453 cells without or with metformin pretreatment. After that, HUVECs were cultured with MDA-MB-453 TCM or the mixture of TCM and supplemented VEGFA (*n* = 6). All data is presented as mean ± S.E.M. *p < 0.05, **p < 0.01, ***p < 0.001.

At present, VEGF is widely thought to be one of the most important angiogenic stimulators in mediating HRG-β1-HER2 signaling induced tumor angiogenesis [18, 19, 22, 23]. To determine the underlying mechanism by which metformin diminished HRG-β1-HER2 signaling-induced promotion of tumor angiogenesis, we detected the change of intracellular expression of VEGF_165_, the major secreted isoform of VEGF [24]. Compared with MDA-MB-231 and MCF-7, VEGF_165_ protein expression was only detected in MDA-MB-453 cell line (Figure 5A and 5B). In comparison with control, HRG-β1 treatment resulted in a great increase of VEGF_165_ in MDA-MB-453 (Figure 5A and 5B), while did not apparently changed the undetectable status of VEGF_165_ in both MDA-MB-231 and MCF-7 cells. Critically, metformin treatment led to a great decrease of both mRNA and protein levels of VEGF even in the presence HRG-β1 (Figure 5B and Supplementary Figure S6). Since VEGF was originally known as a vascular permeability factor inducing intense blood vessel leakage [25], we next studied the effect of metformin on tumor vessel leakage using a 70 kd Fitc-conjugated Dextran. As shown, metformin greatly reduced the amount of dextran leaking outside the tumor vessels (Figure 5C).

To further validate the critical role of VEGF in mediating HER2-induced tumor angiogenesis, bevacizumab (BEV), a specific VEGF neutralizing antibody, and recombinant VEGFA were added to TCM of MDA-MB-453 cells. As shown, BEV greatly inhibited HUVECs proliferation-promoted by the TCM of MDA-MB-453 cells with or not with HRG-β1 pretreatment (Figure 5D). Moreover, the proliferative inhibition-induced by metformin pretreatment was apparently impeded by adding recombinant VEGF (10 ng/ml) to the TCM of MDA-MB-453 (Figure 5E). These data demonstrated the involvement of VEGF in mediating HER2+ tumor angiogenesis and suggest VEGF is an important target of metformin for inhibiting tumor angiogenesis.

### AG825, a HER2-specific inhibitor, abrogated HER2-indcued VEGF expression and reduced microvessel density in HER2^+^ 4T1 tumors

**Figure 6:**
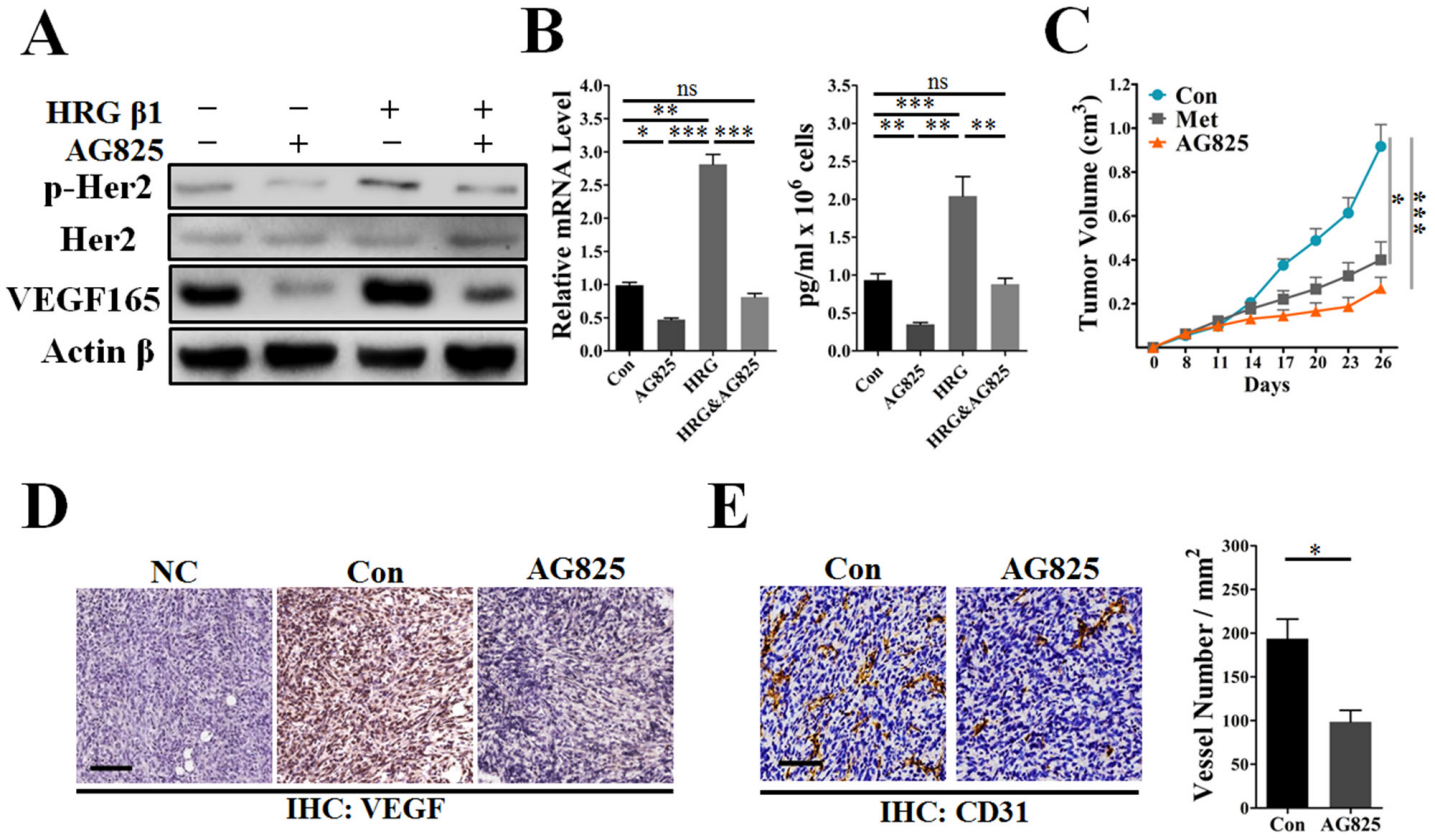
AG825 decreased HER2 signaling-induced VEGF expression and suppressed tumor angiogenesis MDA-MB-453 or 4T1 cells were cultured with metformin (10 mM), AG825 (10 μM) or HRG-β1 (50 ng/ml) in DMEM containing 10% FBS for 24 h, then the intracellular proteins, mRNA and TCM were extracted. **A.** Immunoblotting for the total and phosphorylated levels (Tyr 1221/1222) of HER2 and VEGFA proteins in MDA-MB-453 cells. **B.** Representative images showing the mRNA and secretion levels of VEGF of MDA-MB-453 cells (*n* = 5 for both). **C.** Representative image showing the growth curve of 4T1 tumors from control mice and those treated with metformin (200 mg/kg • day), or AG825 (10 mg/kg. day) (*n* = 6 − 8). **D.** Immunohistochemical staining for VEGFA in 4T1 tumors; scale, 200 μm. **E.** Immunohistochemical staining for CD31^+^ vessels and quantitative measurement of microvessel density in 4T1 tumors (*n* = 6 − 8). Scale bar, 75 μm. All data is presented as mean ± S.E.M. *p < 0.05, **p < 0.01, ***p < 0.001.

As has been well documented, AG825 is a powerful inhibitor of HER2 signaling independent of total HER2 protein expression. To verify our hypothesis that HER2-VEGF signaling was involved in metformin-induced angiogenic suppression, we investigated a possible link between AG825 induced HER2 inhibition and suppressed tumor angiogenesis. As expected, AG825 reduced the phosphorylation level of HER2 while not apparently decreasing its total protein level (Figure 6A). Notably, both mRNA and protein levels of VEGF in MDA-MB-453 were all greatly decreased by AG825 in both the presence and the absence of HRG-β1 (Figure 6A–6B). Compared with metformin treatment, AG825 exhibited similar inhibition of 4T1 tumor growth in BALB/C mice (Figure 6C). Additionally, AG825 treatment decreased the VEGF expression and MVD in the sections of 4T1 tumors (Figure 6D and 6E). These findings indicate that HER2 signaling-induced VEGF up-regulation was associated with a transcriptional regulatory mechanism. Conversely, therapies inhibiting HER2 signaling should block HER2-induced transcriptional activation of VEGF signaling pathway, thus leading to inhibition of angiogenesis in HER2^+^ cancers.

### HIF-1α was required for HER2+ tumor angiogenesis and involved in metformin-induced anti-angiogenesis

**Figure 7:**
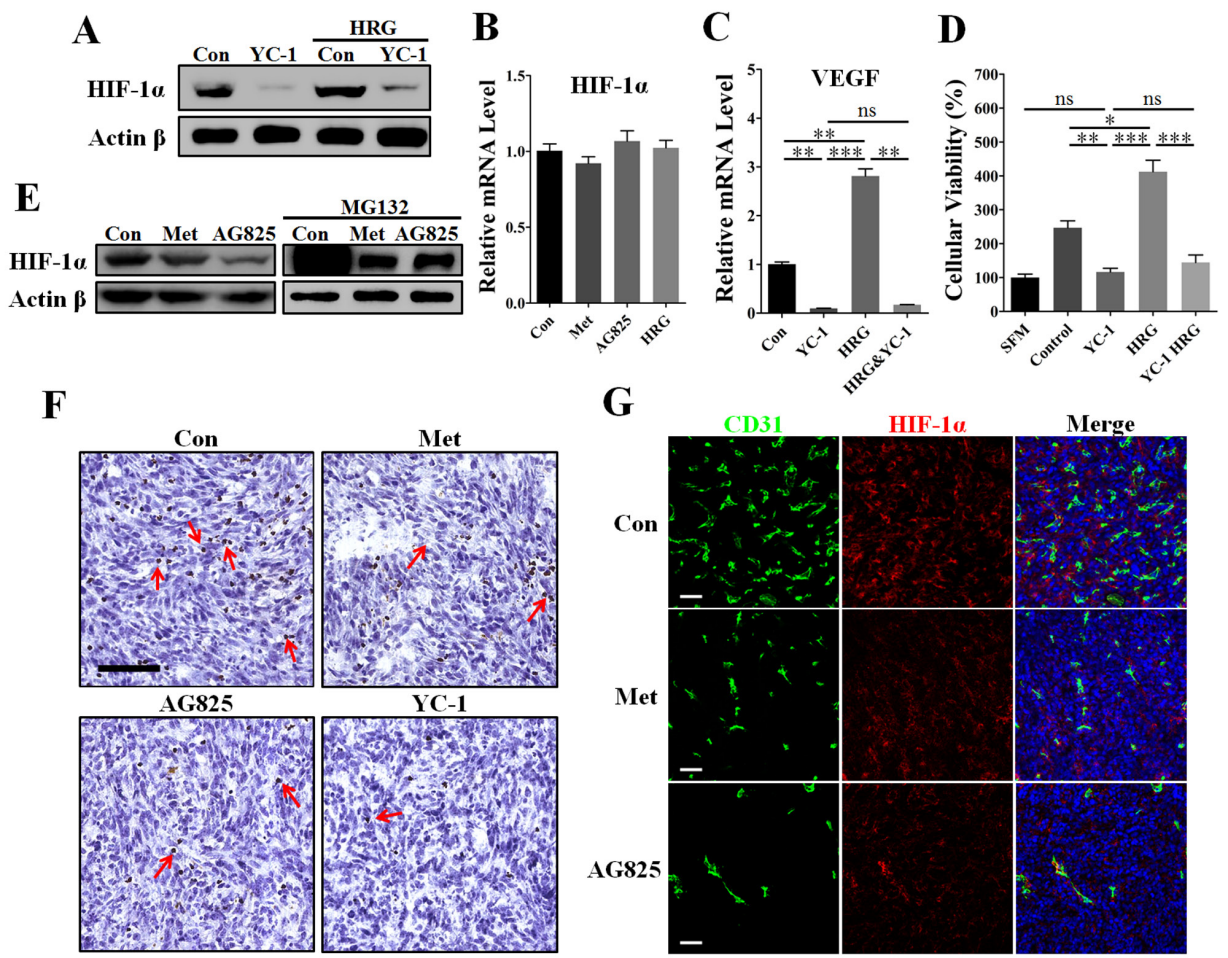
Inhibition of HIF-1α greatly contributed to metformin-induced VEGF down-regulation in the presence of HER2 signaling MDA-MB-453 cells were cultured with metformin (10 mM), AG825 (10 μM), YC-1 (10 μM) or HRG-β1 (50 ng/ml) for 24 h, then the intracellular proteins, mRNA, and tumor cell-conditioned medium (TCM) were extracted. **A.** YC-1 almost completely inhibited HIF-1α expression of MDA-MB-453 cells even in the presence of HRG-β1 treatment. Representative image showing mRNA levels of **B.** HIF-1α and **C.** VEGFA in MDA-MB-453 cells (*n* = 5 for both). **D.** HUVECs were cultured with serum-free medium (SFM) or TCM from MDA-MB-453 cells treated with YC-1 (10 μM), HRG-β1 (50 ng/ml) or both (*n* = 6). **E.** Immunoblotting for protein expression of HIF-1α in MDA-MB-453 cells in both the presence and absence of MG132 (20 μM). **F.** Immunohistochemical staining for HIF-1α in 4T1 tumors from control mice or those treated with metformin (200 mg/kg • day), YC-1 (10 mg/kg. day), or AG825 (10 mg/kg. day). Red arrows indicate the cells with nuclei positive for HIF-1α. Scale bar: 100 μm. **G.** Immunofluorescent double staining for CD31 and HIF-1α in 4T1 tumors. Scale bar: 100 μm. All data is presented as mean ± S.E.M. *p < 0.05, **p < 0.01, ***p < 0.001.

In this paper, we have observed the effect of metformin on decreasing VEGF mRNA abundance in HER2^+^ cancer cells. Since HIF-1α has been generally recognized as a direct transcriptional regulator of VEGF [26], we thus intended to determine whether HIF-1α was involved in metformin-induced VEGF down-regulation through targeting HER2 signaling. To address this, we used the reagent YC-1, a HIF-1α inhibitor [27, 28], and built RNA interference (RNAi) model to further clarify the role of HIF-1α in HER2 signaling-induced VEGF up-regulation. HRG-β1 induced increased HIF-1α and VEGF expressions in MDA-MB-453 cells in normoxia (Figure 7A and Supplementary Figure S7). Notably, YC-1 and RNAi-HIF-1α both greatly diminished the expressions of HIF-1α and VEGF proteins even in the presence of HRG-β1 treatment without affecting HER2 protein expression (Figure 7A and Supplementary Figure S7 and S8). However, the mRNA level of HIF-1α was not apparently affected by AG825, HRG-β1, or metformin treatment (Figure 7B), suggesting a mechanism involving regulation of HIF-1α protein level.

To further confirm the requirement of HIF-1α in HER2 signaling, we performed the tube formation assay using TCM from MDA-MB-453 cells pretreated with RNAi-HIF-1α. Inhibition of HIF-1α by using RNAi or YC-1 both greatly abrogated HRG-β1-induced promotion of angiogenesis (Supplementary Figure S9), thus demonstrating the requirement of HIF-1α for HER2-induced angiogenesis. We then detected the changes of VEGF mRNA level in MDA-MB-453 cells to demonstrate the transcriptional regulation mechanism. As shown in Figure 7C, YC-1 almost completely diminished the mRNA level of VEGF in both the absence and presence of HRG-β1. Additionally, YC-1 pretreatment decreased MDA-MB-453 TCM-promoted HUVECs proliferation to a comparable level of the SFM group, even in the presence of HRG-β1 pretreatment (Figure 7D).

We next focused on the mechanism underlying metformin-induced HIF-1α inhibition by using the reagent MG132, a proteasome inhibitor, and cycloheximide (CHX), a inhibitor of protein generation. Similar to AG825, metformin reduced the protein level of HIF-1α in normoxia (Figure 7E), indicating that metformin–induced down-regulation of HIF-1α involved HER2 signaling. In the presence of MG132, HIF-1α protein expression increased immensely in MDA-MB-453 cells, suggesting that MG132 caused the newly generated HIF-1α to accumulate through blocking proteasome-mediated HIF-1α gradation. Interestingly, both metformin and AG825 induced a similar inhibition of HIF-1α accumulation in the presence of MG132. Furthermore, HER2-HRG-β1 signaling-induced HIF-1α expression was completely abrogated by CHX (Supplementary Figure S10). Together with the evidence that HER2 increased the rate of HIF-1α synthesis [16], our present data support the possibility that metformin slowed down HIF-1α synthesis by targeting HER2 signaling, thus leading to decreased HIF-1α expression.

We next sought to determine whether HIF-1α was implicated in metformin-mediated inhibition of *in vivo* tumor angiogenesis. As shown in Figure 7F, metformin treatment reduced the number of HIF-1α nucleus positive cells in 4T1 tumors, as with AG825 or YC-1. For further investigation, we performed immunofluorescence double staining for CD31 and HIF-1α. In 4T1 tumors from untreated mice, high HIF-1α expression was accompanied by the high microvessel density (Figure 7G and Supplementary Figure S11). Importantly, the HIF-1α fluorescent signal in 4T1 tumors from metformin- or AG825-treated mice was lower than that in tumors from untreated mice, together with the decreased vessel number (Figure 7G). Our results showed the mechanism of HIF-1α-mediated VEGF transcriptional regulation was significantly involved in metformin induced anti-angiogenesis.

## DISCUSSION

Population studies have demonstrated protective activities of the antidiabetic metformin, which is currently the most widely used biguanide, in lowering cancer risk, improving clinical prognosis and enhancing therapeutic efficacy [29–31]. Recently, metformin treatment was shown to induce a significantly better overall and cancer-specific survival outcome of patients only if they had hormone receptor- or HER2-positive tumors [32]. Although metformin treatment might provide a survival benefit when added to systematic therapy [33], the underlying mechanism of metformin targets HER2 signaling was less understood. In the present study, both our *in vivo* and *in vitro* data demonstrated the significant effects of metformin on suppressing HER2 signaling and tumor angiogenesis and growth. Unlike the roles of AG825 and HRG-β1 in affecting HER2 phosphorylation, metformin-induced reduction of HER2 phosphorylation are more likely mediated by decreasing HER2 protein expression. This possibility is supported by the fact that metformin drastically down-regulated HER2 protein levels regardless of the molecular mechanism contributing to the HER2 overexpression [34]. Mechanistically, metformin-induced suppression of HER2 overexpression appears to occur via direct inhibition of p70S6K1 activity, which was demonstrated to be independent of AMPK signaling. In spite of this, whether metformin directly affect the phosphorylation level of HER2 protein needs to be further defined.

One of the important findings is that metformin significantly impaired HRG-β1-induced angiogenic promotion by suppressing the secretion of angiogenic cytokines from tumor cells with high level of phospho-HER2 protein. This effect of HRG-β1 is similar to the re-establishment of HER2 overexpression in cancer cells that endogenously express low levels of HER2 protein [35]. Although HRG does not directly bind to HER2 protein, it can indirectly activates HER2 by promoting the formation of heterodimers with HER3 or HER 4 via transmodulation and transphosphorylation [36]. Notably, HER2 overexpression greatly enhanced the binding affinity of HRG to its receptors by diminishing the rates of ligand dissociation [37]. Conversely, abolishment of HER2 expression on the cell membrane attenuates ligand binding affinity and the functions of HER2 signaling [38], suggesting that HER2 and HRG may strengthen their own signaling function via a mutual complementary mechanism. Based on these finding, it is reasonable to speculate that metformin may accelerate the rates of ligand dissociation by decreasing the protein level of HER2, thus leading to abrogation of the pro-angiogenic effect of HRG-β1-HER2 signaling. However, HRG-β1 can also enhance tumor angiogenesis even in the presence of extremely low HER2 protein level via up-regulation of VEGF [20]. Further study are needed to address whether or how metformin affects HRG-β1-induced angiogenic promotion, even in the absence of HER2 overexpression.

VEGF, a well-characterized angiogenic factor mediating neovascularization in both physiological and pathological conditions, has been mechanistically implicated in HER2 signaling induced abnormal angiogenesis and intense blood vessel leakage in cancers. In light of this discovery, anti-cancer therapies targeting VEGF have been well developed and have shown significant clinical benefits [39]. Indeed, BEV did not completely block the angiogenic promotion-induced by TCM of tumor cells with HER2 overexpression, indicating that there exist other pro-angiogenic factors secreted by tumor cells to promote angiogenesis. Even so, VEGF appears to be the most important factor for mediating HER2 signaling induced angiogenic promotion. In the present study, metformin treatment exerted its inhibitory effect on VEGF expression and secretion, and restrained paracrine VEGF signaling induced tumor angiogenesis. In support of this notion, the vessels in 4T1 tumors from metformin-treated mice were less leaky and had less ability to sprout. Besides, metformin was reported to inhibit VEGF-dependent activation of ERK1/2 in HUVECs [40]. These findings suggest a potential concept that metformin has dual effects on VEGF signaling-mediated tumor angiogenesis: on one hand, metformin decreases the secretion of VEGF from tumor cells by targeting HER2 signaling; on the other hand, metformin has the inhibitory effects on VEGF signaling.

Although HIF-1α is required to transcriptionally regulate VEGF expression [41], it is still controversial whether HIF-1α is directly involved in HER2-induced VEGF expression [16, 42]. Our results of protein expression screening showed a high level of HIF-1α accumulation in HER2^+^ cancer cells, which were accompanied by high VEGF expression. This finding indicates the possible link between HER2 and HIF-1α-VEGF signaling. Decreased HIF-1α protein expression by YC-1, a HIF-1α synthesis inhibitor, almost completely inhibited the mRNA and protein expressions of VEGF, even in the presence of HRG-β1 treatment. However, unlike bevacizumab-induced partial inhibition, YC-1 pretreatment induced an almost complete inhibition of cancer TCM-induced promotion of EC proliferation. The above-mentioned discrepancy also implies that HIF-1α is indispensable for mediating angiogenesis in tumor cells with HER2 overexpression even in normoxia, which is supported by our results related to siRNA for HIF-1α. Interestingly, metformin induced a slightly stronger inhibition of TCM-mediated EC proliferation than bevacizumab, indicating that metformin may also target other tumor cell-derived angiogenic cytokines. Recently, HER2 signaling was reported to increase the synthesis rate of HIF-1α protein and thus enhance VEGF-mediated abnormal tumor angiogenesis. This evidence illustrates the important role of HIF-1α-VEGF signaling in mediating HER2 signaling-induced angiogenesis. In the present study, metformin exerted a similar effects with AG825 on decreasing the protein expression of HIF-1α. Altogether, these evidences suggest that HIF-α is involved in mediating HER2-induced VEGF up-regulation. This is supported by the fact that metformin exerted similar effects with YC-1 and AG825 on decreasing HIF-1α and VEGF expressions, which was accompanied by decreased MVD in 4T1 tumors. However, YC-1 has been demonstrated to decrease the VEGF expression via inhibition of PI3K or NFκB [43], which may act independent of HIF1-α.

The clinical implications of the present findings propose that HER2 was a potential molecular hallmark to predict the anti-angiogenesis response of tumors to metformin treatment. As overexpression of HER2 is frequently detected in various human cancers, our findings are highly relevant to human cancer patients. Since anti-angiogenesis had been demonstrated to be capable of sensitizing tumor cells to chemotherapeutics via normalizing tumor vasculature, our findings thus provide an important implication that metformin may have the potential of remodeling the abnormal tumor vasculature. Since HER2 positive cancer only account for a part of all cancers [45], therefore, further studies should be focused on exploring more molecular hallmarks, which could be significantly targeted by metformin to suppress tumor angiogenesis, and on investigating whether metformin can remodel tumor vasculature and benefit traditional chemo- and radio-therapy.

## MATERIALS AND METHODS

### Cell lines and materials

MCF-10A cells were maintained in phenol red free DMEM/F12 culture medium as suggested by official website of American Type Culture Collection (ATCC). All other cell lines mentioned in the present study were obtained from ATCC and cultured in Dulbecco's modified eagle medium (DMEM) with 10% fetal bovine serum (FBS) in an atmosphere of 5% CO^2^ and 95% room air at 37°C. MG132 and metformin were purchased from Sigma-Aldrich (USA). AG825 and YC-1 were obtained from Cayman company (USA) and the recombinant human HRG-β1 was purchased from PeproTech corporation (USA).

### Animals and 4T1 xenograft model

The experimental protocol was approved by the Ethical Committee and the Institutional Animal Care and Use Committee of Xi'an Jiaotong University. To establish the 4T1 cancer cell xenograft model, 1 × 10^6^ 4T1 tumor cells were subcutaneously injected into the left thigh of 6–8 weeks old female BALB/C mice. According to our observation, the average daily intake of drinking water of each mouse was about 3 mL. Nine days after the transplantation, the mice were randomly divided into four groups (*n* = 6). BALB/C mice in the control group were intraperitoneally (i.p.) injected with 0.1 ml phosphate-buffered saline (PBS) daily. Animals in treatment groups were injected i.p. with AG825 at a dose of 10 mg/kg or YC-1 at a dose of 10 mg/kg day. For the metformin treatment group, metformin (200 mg/kg • day) was added to the drinking water. It was observed that metformin treatment induced no significant difference in the intake of drinking water. The tumor volumes were measured every two or three days by using a caliper and calculated by the following formula: length × width^2^ × 0.523. Since severe organ dysfunction related to distant metastasis will interfere with our observation of tumor growth, the xenograft experiment was thus terminated when average tumor volume of the untreated group reached 1 cm^3^. All animals were sacrificed by injecting 90 μl ethyl carbamate (20%) into the abdominal space, and tumors were integrally removed and measured by an electronic balance.

### Preparation of tumor cell-conditioned medium

To extract tumor cell-conditioned supernatants, 1 × 10^6^ tumor cells were inoculated in 6 cm petri dishes and cultured in 10% FBS DMEM supplemented with metformin, HRG-β1, YC-1, AG825 or the combined pretreatment for 24 h. Then tumor cells were washed three times with PBS and subsequently cultured in SFM for another 24 h. Following the inoculation period, TCM was collected and centrifuged at 500 × g to remove detached cells and then at 12,000 g to discard cell debris (4°C, 10 minutes each). Each corresponding dish was subsequently trypsinized, and the number of live cells was counted to allow an appropriate correction of TCM loading for cell equivalents.

### Co-culture vascular sprout assay

To mimic the *in vivo* angiogenesis, we used a cell culture insert with polycarbonate membrane (pore size: 0.4 μm, Thermo Scientific Nunc, USA). This insert was pre-packed in a 6-well multidish. 20,000 HUVECs and 40,000 tumor cells were respectively seeded onto the bottom of the well and the inside surface of the insert. All the cells were further cultured with SFM for 24 h. After that, the insert and culture medium were removed, and HUVECs were washed three times with PBS. Then, HUVECs were fixed by 4% paraformaldehyde (PFA) and stained with haematoxylin and esion (H&E). Five random fields of each well were subsequently recorded by a light microscopy (Leica, German). Finally, the number and length of the sprout per HUVEC was quantified and statistically analyzed.

### *In vitro* tube formation assay

100 μL Matrigel Basement Membrane Matrix (BD Biosciences, USA) was used to precoat the bottom of a 96-well plate for polymerization at 37°C for 30 minutes. Then, 20,000 or 12,000 HUVECs were seeded into each well in SFM or 75% TCM. After incubation for 12 h or 24 h at 37°C, a capillary-like network was formed and the pictures were recorded by an upright microscope. Finally, the length of the vascular network was measured by using ImageJ 2x software (NIH, Bethesda, MD).

### Cell proliferation assay

CCK-8 (Cell counting kit-8, DoJinDO, Japan) assay was performed to observe the changes of HUVECs viability following the protocol. The final absorbance of each well was determined at 450 nm using a micro-plate reader.

### Huvecs invasion assay

For invasion assays, the membrane surface of Transwell (pore size: 8 μm, Corning, USA) was pre-coated with Matrigel (BD Biosciences) and incubated at 37°C for 30 min. HUVECs (50,000 in DMEM with 2.5% FBS) were inoculated onto the upper chambers, and the bottom chambers were filled with the mixture of 75% TCM and 25% DMEM supplemented with 2.5% FBS. HUVECs were allowed to invade for 10 h at 37°C and 5% CO_2_. After scraping the cells on the top surface, the invaded cells were fixed with 4% PFA for 1 h and washed for three times with PBS, followed by staining with crystal violet and air dry at room temperature. Images were finally taken using a Leica inverted micro-scope.

### Quantitative real-time pcr and western blotting

The quantitative Real Time PCR was performed to determine the changes of VEGF and HIF-1α mRNA levels. The primer sequences used for amplifications were as follows (5` to 3`): HIF-1α: AGT GGTATTATTCAGCACGAC (forward), AAGGCAGC TTGTATCCTCT (reverse); VEGF: CATGGATG TCTACCAGCGAA (forward), CCAGGATTTAAACC GGGAT (reverse); HER2: TCCATCATCTCTGCGGTGGT (forward), CAGCAGTCTCCGCATCGTGT (reverse).

To detect the protein levels, the equal amount of protein was separated by SDS-PAGE and transferred to polyvinyldifluoride membranes (Millipore, USA). Then membranes were incubated at 4°C overnight with antibodies, including anti-HIF-1α antibody (Abcam, USA), anti-VEGF antibody (Proteintech), anti-HER2 antibody (Sangon, China) and anti-p-HER2 (Phospho-Tyr1221/Tyr1222) antibody (Sangon, China), and followed by incubation with goat anti-rabbit or anti-mouse IgG (H+L) conjugated with horseradish peroxidase. Band intensity was finally determined using ImageJ 2x software.

### Immunohistochefmical and immunofluorescent analysis

4T1 tumor specimens were embedded into paraffin and subsequently cut into 4 μm-thick sections. To performing the morphological analysis, tumor sections were immunolabeled with the HIF-1α (1:200), VEGF (1:400), EC-marker CD31 (Abcam, 1:100) and p-HER2 (1:400).

For fluorescent analysis, frozen tumor samples were cut into 8 μm-thick sections with cryostat. Tumor vessels were immunolabeled with rabbit anti-CD31 antibody (1:40) and followed by the goat anti-rabbit antibody conjugated with Alexa Fluor 488 (Life Tech, USA). 3D reconstruction was performed to investigate the change of vascular branches. Tumor sections from different groups were also incubated with HIF-1α antibody (1:50) and followed by the donkey anti-mouse antibody conjugated with Alexa Fluor 546 (Life Tech, USA). Double stained sections for CD31 and HIF-1α were recorded using fluorescence microscopy (Leica, German).

### Determination of vascular leakage

To study functional impacts of metformin on vessel leakage of 4T1 tumors, 100 mg/ml Fitc-conjugated Dextran (70 kD; Sigma) in 100 μl was injected intravenously via the tail vein into BALB/C mice. After 30 min to allow circulation, mice were euthanized by cervical dislocation and 4T1 tumors were removed without perfusion. Then, tumors were allowed to be fixed in 4% PFA for 8 h and subsequently immersed in 30% sucrose until all the tumors sank to the bottom of the container. Then the solid tumors were embedded in OCT and 6 μm sections were cut. The extent of Fitc-dextran leakage from blood vessels was estimated on the basis of green fluorescence located external to CD31-positive (Red, TRITC-conjugated) vessels.

### Statistical analysis

All data are expressed as mean value ± SEM (standard error of the mean) from at least three independent experiments. The difference between groups was analyzed using Student's *t* test when only two groups were compared or by one-way ANOVA analysis when three or more groups were compared. All statistical tests were two-sided. Differences were considered statistically significant at *P* < 0.05. All analyses were performed using the software of GraphPad Prism 5.

## SUPPLEMENTARY INFORMATION OF MATERIALS AND METHODS, FIGURES



## Abbreviations

ADA: American diabetes association
ATCC: American type culture collection
AMPK: AMP-activated protein kinase
CHX: cycloheximide
DMEM: Dulbecco`s modified Eagle`s medium
HER: human epidermal growth factor receptor
ECs: endothelial cells
EGFR: epidermal growth factor receptor
ER: estrogen receptor
FBS: fetal bovine serum
HIF-1α: hypoxia inducible factor 1α
HRG or HRG-β1: heregulin or heregulin-β1
HMEC-1: human microvascular endothelial cell
HUVEC: human umbilical vein endothelial cell
mTOR: mammalian target of rapamycin
PBS: phosphate-buffered saline
PFA: Paraformaldehyde
PR: progesterone receptor
RNAi: RNA interference
T2D: type 2 diabetes
TCM: tumor cell-conditioned medium
SFM: serum-free medium
VEGF: vascular endothelial growth factor
