# Supplementary material for: Suppression of tumor angiogenesis by metformin treatment via a mechanism linked to targeting of HER2/HIF-1α/VEGF secretion axis
Source: Oncotarget. 2015 Nov 2;6(42):44579–92. doi: 10.18632/oncotarget.6373 (PMC4792577; doi:10.18632/oncotarget.6373)
Supplement: Supplementary file 1 [file oncotarget-06-44579-s001.pdf]

SUPPLEMENTARY INFORMATION OF MATERIALS AND METHODS

RNA Interference

To perform the RNA interference experiment, we purchased the siRNA for HIF-1α from GenePharm (Shanghai, China). 3x105 MDA-MB-453 cells were seeded in a six-well plate and transfected with 100 nM siRNA by using lipofectamine RNAi MAX reagent (Invitrogen, USA) according to the manufacturer's instructions. The related sequences were listed as following:

SiRNA for HIF-1α: 5′-CCACCACUGAUGAAUUAAATT-3′, 5′-UUUAAUUCAUCAGUGGUGGTT-3′

Negative Control siRNA: 5′-UUCUCCGAACGUGUCACGUTT-3′, 5′-ACGUGACACGUUCGGAGAATT-3′)

SUPPLEMENTARY FIGURES

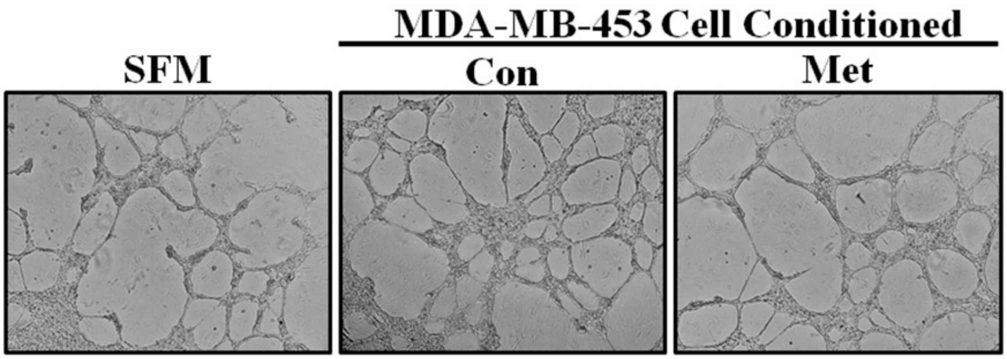

Supplementary Figure S1: Representative images of HMEC-1-mediated tube formation. HMEC-1 was cultured in the presence of SFM or 75% TCM of MDA-MB-453 cells either, with or without the pretreatment of metformin. 200X.

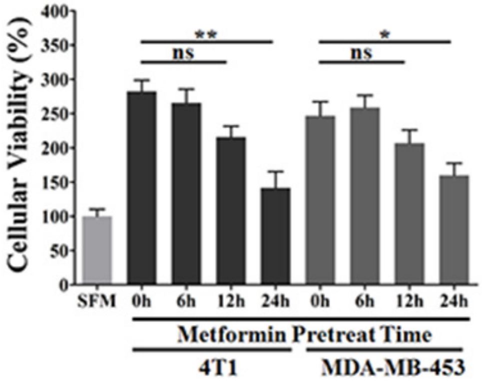

Supplementary Figure S2: Representative images of HUVEC proliferation promoted by TCM of MDA-MB-453 cells that were pretreated with metformin over multiple time periods ($n = 6$).

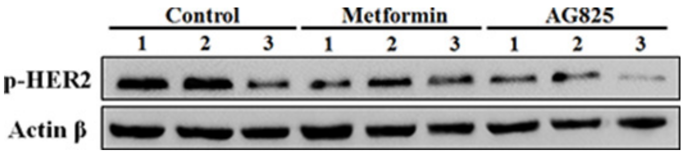

Supplementary Figure S3: Immunoblotting for p-HER2 (Tyr 1221/1222) and actin-β in 4T1 tumors from mice treated with metformin (200 mg/kg day), AG825 (10 mg/kg day), or feeding with water (control). Equal amounts of total proteins (80 μg/lane) were loaded.

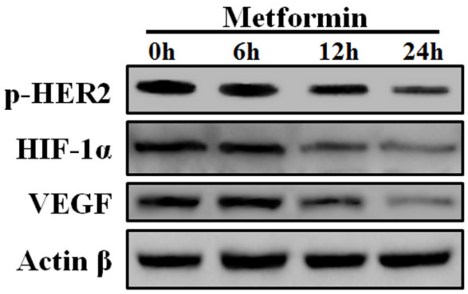

Supplementary Figure S4: Immunoblotting for detection of protein levels of phospho-HER2, HIF-1α, and VEGF in MDA-MB-453 cells treated with metformin over multiple time periods.

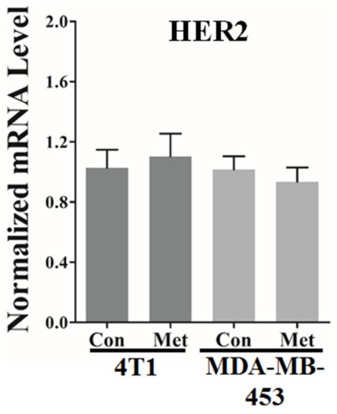

Supplementary Figure S5: Representative images of the mRNA levels of HER2 in 4T1 and MDA-MB-453 cancer cells treated with metformin or not (n = 5, n = 5, respectively).

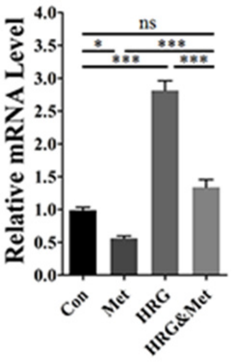

Supplementary Figure S6: Representative images showing the mRNA level of VEGF in MDA-MB-453 cells (n = 5).

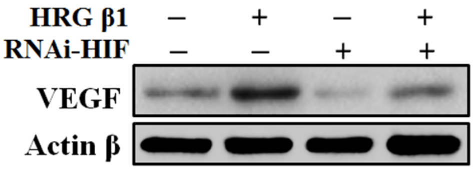

Supplementary Figure S7: Immunoblotting for detecting protein levels of $VEGF_{165}$ in MDA-MB-453 cells.

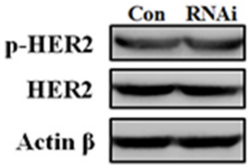

Supplementary Figure S8: Immunoblotting for total level and phosphorylation level of HER2 protein in MDA-MB-453 cells.

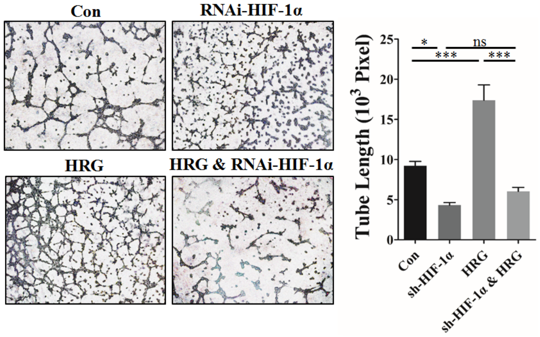

Supplementary Figure S9: Representative images showing HUVEC-mediated tube formation. HMEC-1 was cultured in the presence of SFM or 75% TCM from MDA-MB-453 cells transduced or not transduced with RNAi targeting HIF-1α in the absence or presence of Heregulin (HRG)-β1 pretreatment. 200X (Left). Quantification of the tube length produced by HUVECs (Right).

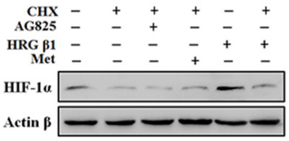

Supplementary Figure S10: Immunoblotting for the detection of the protein level of HIF-1α in MDA-MB-453 cells.

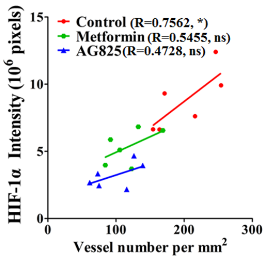

Supplementary Figure S11: Representative images showing the linear correlation between HIF-1α fluorescent intensity and microvessel density in 4T1 tumor. Sperman analysis was used to determine the significance of the linear correlation. *$P < 0.05$.
